# Mental disorders in adults deprived of liberty in American countries: a scoping review

**DOI:** 10.1590/0102-311XEN200624

**Published:** 2025-10-03

**Authors:** Glaucia Mayara Niedermeyer Orth, Fernanda Serpeloni, Simone Gonçalves de Assis, Thais Afonso Andrade, Elisa Maria Rabe, Annelise Aurea Araújo de Moura

**Affiliations:** 1 Defensoria Pública do Estado do Paraná, Curitiba, Brasil.; 2 Escola Nacional de Saúde Pública Sergio Arouca, Fundação Oswaldo Cruz, Rio de Janeiro, Brasil.; 3 Centro Universitário Senai CIMATEC, Salvador, Brasil.; 4 Prefeitura Municipal de Castro, Castro, Brasil.; 5 Universidade Federal do Rio de Janeiro, Rio de Janeiro, Brasil.

**Keywords:** Mental Health, Global Health, Mental Disorders, Prisons, Saúde Mental, Saúde Global, Transtornos Mentais, Prisões, Salud Mental, Salud Global, Trastornos Mentales, Prisiones

## Abstract

A large proportion of the world’s incarcerated population is concentrated in the Americas. Research has consistently shown that mental health disorders are more prevalent among incarcerated individuals compared to the general population. Despite this, mental health necessities in prisons are often neglected, and in the Americas, the treatment gap for mental disorders remains substantial. This study aimed to conduct a scoping review of the scientific literature on the prevalence of mental health problems among incarcerated populations in American countries, from a global mental health perspective. The search included databases such as SciELO, Embase, PubMed/MEDLINE, Web of Science, Scopus, PsycINFO, and CINAHL (EBSCO) and covered publications from 2013 to 2023 in Portuguese, English, and Spanish. The final sample comprised 42 articles, of which South American countries were the most studied, followed by North American countries, while Central America had the least representation. The focus of the studies were as follows: the prevalence of mental health problems among incarcerated populations in the Americas; the links between mental disorders, aggressive behavior, and criminal recidivism; mortality among incarcerated individuals; and stigma as a barrier to accessing mental health services. According to the outcomes, investing in mental health services is essential to provide adequate care to prisoners, which could help reduce incarceration rates. However, overcrowding in prisons across the continent poses a significant barrier to the provision of mental health treatments.

## Abstract (pt)

Uma parcela significativa da população encarcerada no mundo está concentrada nas Américas. Há evidências consistentes de que transtornos mentais são mais prevalentes entre os indivíduos encarcerados em comparação à população em geral. Apesar disso, as necessidades de saúde mental nas prisões são frequentemente negligenciadas. Ademais, nas Américas, a lacuna no tratamento de transtornos mentais permanece substancial. O objetivo deste estudo foi conduzir uma revisão de escopo da literatura científica sobre a prevalência de problemas de saúde mental entre populações encarceradas em países americanos sob uma perspectiva global de saúde mental. A busca foi realizada em bases de dados, como SciELO, Embase, PubMed/MEDLINE, Web of Science, Scopus, PsycINFO e CINAHL (EBSCO), para artigos publicados entre 2013 e 2023 em português, inglês e espanhol. A amostra final foi composta por 42 artigos. Os países da América do Sul foram os mais estudados, seguidos pelos países da América do Norte, enquanto a América Central teve a menor representatividade. Os estudos analisados focaram em: a prevalência de problemas de saúde mental entre as populações encarceradas nas Américas; as ligações entre transtornos mentais, comportamento agressivo e reincidência criminal; mortalidade entre indivíduos encarcerados; e o estigma como barreira ao acesso a serviços de saúde mental. Constatou-se que investir em serviços de saúde mental é essencial para fornecer atendimento adequado aos presos, o que poderia ajudar a reduzir as taxas de encarceramento. No entanto, a superlotação nas prisões em todo o continente representa uma barreira significativa à prestação de tratamentos para a saúde mental.

## Abstract (es)

Una parte significativa de la población carcelaria mundial se concentra en América. Investigaciones han demostrado sistemáticamente que los trastornos de salud mental son más frecuentes entre las personas encarceladas que entre la población general. A pesar de ello, las necesidades de salud mental en las prisiones suelen descuidarse y, en América, la carencia de tratamiento de los trastornos mentales sigue siendo considerable. El objetivo de este estudio fue realizar una revisión exploratoria de la literatura científica sobre la prevalencia de los problemas de salud mental entre la población reclusa en los países americanos, desde una perspectiva de salud mental global. La revisión incluyó bases de datos como SciELO, Embase, PubMed/MEDLINE, Web of Science, Scopus, PsycINFO y CINAHL (EBSCO) y abarcó publicaciones de 2013 a 2023 en portugués, inglés y español. La muestra final comprendió 42 artículos, siendo los países sudamericanos los más estudiados, seguidos por los países norteamericanos, mientras que Centroamérica tuvo la menor representación. Los estudios examinados se centraron en: la prevalencia de los problemas de salud mental entre la población reclusa en las Américas; los vínculos entre los trastornos mentales, el comportamiento agresivo y la reincidencia delictiva; la mortalidad entre las personas reclusas; y el estigma como barrera para acceder a los servicios de salud mental. Se ha constatado que invertir en servicios de salud mental es esencial para proporcionar una atención adecuada a los reclusos, lo que podría contribuir a reducir las tasas de encarcelamiento. Sin embargo, el hacinamiento en las prisiones de todo el continente supone un obstáculo importante para la prestación de tratamientos de salud mental.

## Introduction

Approximately 11 million people are incarcerated worldwide. Globally, this population is unevenly distributed across continents. Asia holds the largest prison population, estimated at 4,316,992 individuals, followed by the Americas with 3,730,016, Europe with 1,496,614, Africa with 1,384,534, and finally Oceania with 63,177 incarcerated people. A large proportion of the world’s incarcerated population is concentrated in the Americas, which also has the highest incarceration rate globally, at 357 prisoners per 100,000 of the national population. Asia, despite having the largest prison population, has a lower incarceration rate of 95 per 100,000 people ^1^. Of the incarcerated population worldwide, more than 740,000 are women and girls, representing less than 7% of the global prison population. Asia experienced the largest increase in female prisoners, with numbers more than doubling since the early 2000s. American countries such as El Salvador, Guatemala, and Brazil have observed significant rises in their female prison populations ^2^. Despite this growth, men remain the majority within the prison environment.

Prison overcrowding is a global issue, occurring in varying degrees across all continents ^3^. The United States has the world’s largest incarcerated population, with 1,767,200 individuals in detention. Brazil ranks third globally, with 839,672 incarcerated people. However, prison conditions in North America differ significantly from those in Central and South America. Despite having the highest number of incarcerated individuals, the United States does not experience overcrowding in its facilities, contrasting with many countries in Central and South America. South America alone has a prison population of 1,376,530, and since 2000, the total number of incarcerated individuals has tripled, making it the region with the highest increase in prison population globally ^1^.

The highest rates of violence worldwide are found in the Americas, with Latin America and the Caribbean holding the highest homicide rates, which is related to high drug production and trafficking, proliferation of criminal organizations and the use of firearms ^4^. Furthermore, homicide clearance rate by the police forces is low: for every 10 homicide victims, fewer than two suspects are convicted across the American continent ^4^. While there are significant regional variations, organized crime - including drug trafficking and gang violence - is a primary driver of homicides, with youth disproportionately involved both as victims and perpetrators ^4^. Despite low homicide case resolution rates, North and South America house the largest prison populations on the continent, whereas Central America has the highest incarceration rate globally ^1^. Violence continues within the prison system as well, where drug trafficking organizations, militias, and gangs infiltrate overcrowded prisons, perpetuating cycles of violence ^4^.

In the *World Report on Violence and Health* ^5^, published in 2002, the World Health Organization (WHO) recognized violence as a major public health issue requiring preventive action on a global scale. Populations exposed to violence are vulnerable to injuries, death, and other detrimental impacts on their physical and mental development. Additionally, violence serves as a risk factor for numerous social issues, which is why violence reduction is among the Sustainable Development Goals (SDGs) of the United Nations ^6^.

Research consistently shows a significantly higher prevalence of mental disorders and substance use among incarcerated populations compared to the general public ^7^ ^,^ ^8^ ^,^ ^9^ ^,^ ^10^. Mental disorders are clinically significant conditions characterized by disruptions in thought, mood, and behavior, which lead to functional impairments in one or more areas of life and are present across various social classes and gender relations ^11^. Incarcerated individuals experience a substantial burden of mental health disorders, at least twice as prevalent than community dwellers: major depression (11.4%), post-traumatic stress disorder (PTSD) (9.8%), psychotic illness (3.7%), alcohol use disorder (23.8%), and drug use disorder (38.9%) ^12^. For instance, drug use and PTSD were more prevalent in women than in men, and psychotic illness and depression were more common in low- and middle-income countries compared to high-income countries ^10^.

Besides showing a higher prevalence of mental disorders and substance use, the mental health necessities of the incarcerated population are frequently neglected, often underdiagnosed and untreated ^13^. Despite the greater need for care, prisons are regarded primarily as spaces for punishment.

There are significant treatment gaps for the incarcerated population in need of mental health care, which remains a relevant and current global health issue ^14^ ^,^ ^15^. In the Americas, mental and substance use disorders represent pressing public health challenges, with the global mental health burden estimated at 10.5% to 19%. Additionally, the treatment gap for any mental disorder in the continent reaches 71.2%, with the highest gap found in Mesoamerica at 77.4%, including countries like Mexico, Guatemala, El Salvador, Honduras, Costa Rica, Nicaragua, and Panama. Substance use disorders represent the largest treatment gap in the Americas, estimated at 83.7% in Latin America and 69.1% in North America ^16^.

Such gaps are influenced not only by poor investments in mental health services but also by individual perceptions regarding the need to seek treatment ^17^. The economic and social contrasts within the Americas, ranging from high-income countries to those with lower socioeconomic development and varying levels of income inequality (Gini coefficient), impact on the organization of mental health services. According to the *Mental Health Atlas* (2020) ^18^, a positive correlation between mental health service investment and national gross income exists, which means high-income countries invest more in mental health. Consequently, the treatment gap for severe mental disorders is lower in North America (40.5%) compared to other regions in the Americas, although it remains substantial ^16^.

There are effective treatments for most mental disorders, yet the unequal distribution of financial resources between countries (high-income vs. low- and middle-income countries) hinders the universal provision of mental health treatments to those in need, and this disparity represents a critical issue in global mental health ^19^. Mental disorders are the leading cause of disability, and individuals with severe mental health conditions face premature mortality from preventable physical diseases. These circumstances yield negative economic impacts on countries, underscoring why mental health is a global concern ^20^.

Given the observed treatment gaps in mental health across the Americas, high violence rates, and the relevance of mental disorders among the incarcerated population, this article aims to conduct a scoping review of scientific literature concerning mental health disorders within incarcerated populations across American countries with varying levels of economic and social development. This review is framed within a global mental health perspective.

## Method

A scoping review was conducted on the scientific literature regarding mental health disorders in incarcerated populations in countries across the American continent. The development of this review followed the guidelines of the PRISMA-ScR tool (*Preferred Reporting Items for Systematic Reviews and Meta-Analyses: Extensionj for Scoping Reviews*) ^21^ and adhered to the methodology outlined in the Joanna Briggs Institute (JBI) manual ^22^. The review protocol was registered on the Open Science Framework platform (https://osf.io/248tf/).

The study was guided by the research question: “What is the scope of scientific literature on mental health disorders in incarcerated populations in American countries?”. The research question, as recommended by the JBI methodology, was structured using the PCC (population, concept, and context) strategy, respectively: adults in incarceration, mental health disorders, and the countries across the American continent.

### Search strategy and eligibility criteria

The initial inclusion criteria were studies published between 2013 and May 2023, with any methodological design focusing on mental health disorders in incarcerated adults within the American continent, published in English, Portuguese, or Spanish. Gender was not an exclusion criterion. The initial exclusion criteria included publications in books, book chapters, academic thesis and dissertations, monographs, and studies published in languages other than those listed, as well as studies that did not address the topic of mental health disorders in incarcerated populations within the American continent.

Searches were conducted in May 2023, covering publications from 2013 onwards, in the following databases: SciELO, Embase, PubMed/MEDLINE, Web of Science, Scopus, PsycINFO, and CINAHL (EBSCO). The search strategy employed the following keywords: *“Incarcerated” OR “Prisoner” OR “Prison” OR “Jails” OR “Penitentiary” OR “Detention”) AND (“Aggression” OR “Aggressive” OR “Psychiatric Disorders” OR “PTSD” OR “Panic Disorders” OR “Panic Attack” OR “Suicidal” OR “Suicide” OR “Depression” OR “Anxiety” OR “Phobia” OR “Selfharm” OR “Alcohol” OR “Substance Abuse*”.

Article selection was conducted independently by two authors and cross-checked by a third reviewer based on the defined inclusion and exclusion criteria. After duplicate entries were removed, titles and abstracts were screened using the bibliographic management tool Rayyan (https://www.rayyan.ai/) ^23^. Discrepancies were resolved by consensus and studies focusing on incarcerated migrants, research on mental disorders developed after incarceration, and review articles were excluded.

### Data extraction and analysis

Following the reading of titles and abstracts, and the selection of eligible studies, these were exported into an Excel spreadsheet (https://products.office.com/), in which the following data were extracted based on the full text of the articles: authors; title; publishing journal; complete reference including DOI; abstract; language of the article; year of publication; research objective; methodology used; age range/target audience; gender of participants; country of study; key findings; types of mental disorders studied; prevalence of mental disorders; pre-incarceration vulnerabilities/violence and risks during incarceration, and whether the article addressed any physical health issues. Data extraction after full reading of the articles was conducted by the reviewers. Decisions were made jointly by two or more authors when new exclusions were necessary.

**Figure 1:**
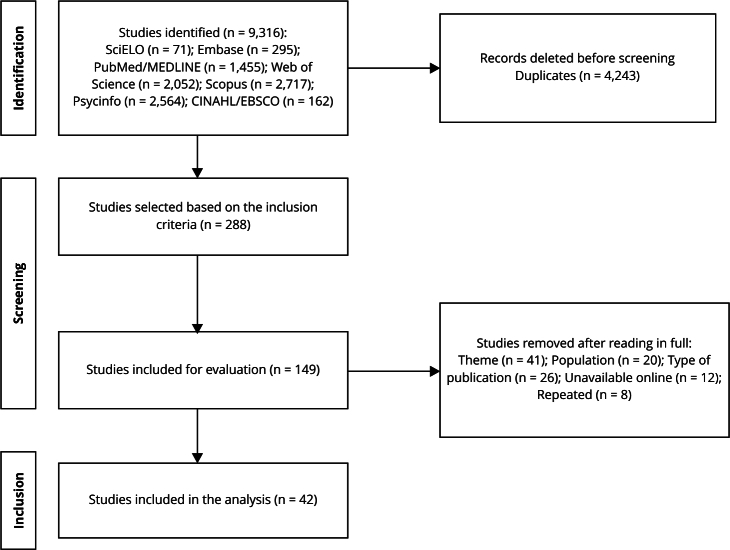
Flow diagram.

Source: prepared by the authors, based on PRISMA statement (2020) ^84^.

Of the 9,316 records initially identified in the search, 4,243 duplicates were excluded prior to screening. Of the remaining 5,073 records, 288 studies were selected, applying the geographic criterion (American continent). Of these, 149 studies were deemed eligible following a double-blind screening for inclusion and exclusion criteria via title and abstract review. Then, a full-text review of the 149 selected studies. From these, 107 were excluded for not meeting the research criteria, such as lacking a focus on mental health, not involving an adult incarcerated population, unavailability in electronic format, non-compliant publication types, and duplicates (same study with titles in different languages). Figure 1 shows the flow diagram for this scoping review.

The 42 selected publications were analyzed according to two analytical approaches.

(1) Characterization of the collection: this involved a data matrix describing the publications by title, authorship, abstract, country of research and country of the authors, year of publication, target age group, gender, types of mental disorders studied, prevalence of mental disorders, pre-incarceration vulnerabilities/violence, risks during incarceration, and the level of economic development ^24^. The level of economic development of American countries was based on gross national income (GNI) per capita, which is organized by the World Bank’s classifications (2024): low-income countries (USD 1,145 or less); low- and middle-income countries (between USD 1,146 and USD 4,515); upper-middle-income countries (between USD 4,516 and USD 14,005); and high-income countries (more than USD 14,005) ^25^.

(2) Thematic grouping: based on content analysis using a thematic approach, the publications were grouped according to the similarity of their topics and the focus on global mental health ^26^. The following categories emerged: the importance and prevalence of mental health problems, prison mortality, violent behavior/recidivism, and stigma.

## Results

### General profile

The countries researched were distinguished from the countries of the authors. Regarding the number of countries investigated in the publications, 57.1% represented South America, led by Brazil, followed by Chile, Colombia, Ecuador, French Guiana, Argentina, and Peru. North America accounted for 38.1% of the studies, of which Canada was the leader, followed by the United States and Mexico. Lastly, Central America represented 4.8%, with two publications on Grenada. Note that Central America had the lowest participation in publications on mental disorders within the prison system based on the scope of this analysis.

When examining the countries of authors, North America (47.6%) and South America (40.4%) had similar proportions, while the remainder originated from European countries (Germany, England, the Netherlands, and Spain) and Australia. Notably, there were no authors from Central America.

Regarding economic levels, 52% of the publications came from upper-middle income countries, 45.3% from high-income countries, and 4.7% from a country without economic development information. There were no publications from low-income or lower-middle income countries.

Most articles were written in English (66.7%), followed by Spanish (23.8%) and Portuguese (9.5%). Regarding publication years, 2014 (6 articles), 2016 (9 articles), and 2017 (6 articles) had the highest frequencies, with a gradual decline in recent years. Most articles considered a mixed-gender target audience, including both men and women (52.3%), while 35.7% were male-focused, and 7.1% female-focused. One study included a transgender females, while another did not specify a gender focus.

The methodologies were mostly quantitative, based on epidemiological studies: descriptive (primary and/or secondary data) ^27^ ^,^ ^28^ ^,^ ^29^ ^,^ ^30^ ^,^ ^31^ ^,^ ^32^ ^,^ ^33^ ^,^ ^34^, cross-sectional ^35^ ^,^ ^36^ ^,^ ^37^ ^,^ ^38^ ^,^ ^39^ ^,^ ^40^ ^,^ ^41^ ^,^ ^42^ ^,^ ^43^ ^,^ ^44^ ^,^ ^45^ ^,^ ^46^ ^,^ ^47^ ^,^ ^48^ ^,^ ^49^ ^,^ ^50^ ^,^ ^51^ ^,^ ^52^ ^,^ ^53^, longitudinal/cohort ^54^ ^,^ ^55^ ^,^ ^56^ ^,^ ^57^ ^,^ ^58^ ^,^ ^59^, time series analysis ^60^, case-control ^61^, among others. Mixed methods research ^62^ ^,^ ^63^ and action research ^64^ were also found, and there were no articles with qualitative research methodology.

### Overview of incarceration in the Americas

American countries have significant regional differences in terms of economic, political, social, and cultural development. The publications identified primarily come from upper-middle-income and high-income countries, focusing on North and South America. Central America, which was represented in only two studies (both from Grenada), stands out with the highest incarceration rate globally (310.5 per 100,000) ^1^. Research on the penitentiary system in this region often lacks a focus on mental health, possibly highlighting the invisibility of mental disorders related to incarceration and the predominance of State punishment as the main approach to combat violence. An example is the “La Mano Dura” policies in Guatemala ^65^, El Salvador ^66^, and Honduras ^66^, which reflect the aggressive stance of the Central American countries against gangs and drug trafficking.

The countries with the largest prison populations in the Americas are those located in the northern and southern extremes, where the largest territories and populations are concentrated. Notably, despite having a relatively small prison population with an occupancy rate of 102.2%, Canada has the highest number of publications on the mental health of incarcerated populations (11 articles). Additionally, since 2016, Canada’s prison population has decreased ^1^, and its correctional system emphasizes restorative justice that places a significant focus on offender rehabilitation ^67^. This emphasis likely contributes to the high level of interest in the mental health of incarcerated individuals within Canadian research.

Box 1 shows the distribution of texts found in the review, categorized by the countries’ economic development, which is organized by region (Northern America, Central America and the Caribbean, and South America), prison population and occupancy level of each country, including World Bank classification of the countries, and the mental health disorders investigated. Also, regarding the number of articles from each country, in South America, most part of the articles came from upper-middle income countries (Brazil, Colombia, Ecuador, Argentina, Peru) and in Northern America, the most studies were from high-income countries (USA and Canada).

**Box 1:** Countries in the Americas by region, economic development pattern, prison population/overcrowding and mental health disorders investigated in the review.

REGION	COUNTRY	PRISON POPULATION *	OCCUPANCY RATE (%) **	WORLD BANK COUNTRY CLASSIFICATION ***	MENTAL HEALTH DISORDERS INVESTIGATED
North America	United States	1,767,200	95.6	High income	Alcohol and drug use, PTSD, suicide, depressive-anxious (3 articles)
	Canada	35,485	102.2		ADHD, drug use, depression, psychosis, PTSD, anxiety, personality disorder, cognitive deficit, bipolar disorder, non-suicidal self-injury (11 articles)
	Mexico	231,954	103.9	Upper-middle income	Depression, alcohol and drug use (2 articles)
	Subtotal	2,034,639			
Central America and the Caribbean	Bermuda	124	59.7	High income	-
	Panama	23,798	163.1		-
	Antigua and Barbuda	400	266.7		-
	Bahamas	1,617	161.7		-
	Barbados	743	61.5		-
	St. Kitts & Nevis	160	144.0		-
	Trinidad & Tobago	3,802	81.8		-
	Aruba	311	68.4		-
	Cayman Islands	226	122.8		-
	Curaçao	429	63.9		-
	Puerto Rico	7,176	69.7		-
	Sint Maarten	87	96.3		-
	United States Virgin Islands	359	162.5		-
	Belize	1,199	57.1	Upper-middle income	-
	Costa Rica	17,829	120.4		-
	El Salvador	71,000	236.7		-
	Guatemala	23,361	299.4		-
	Cuba	90,000	NI		-
	Dominica	261	73.0		-
	Dominican Republic	25,280	161.0		-
	Grenada	385	194.4		Drug use, personality disorder, depression, anxiety, bipolar disorder, psychosis (2 articles)
	Jamaica	3,559	87.0		-
	St. Lucia	572	102.8		-
	St. Vincent & the Grenadines	392	79.8		-
	Honduras	19,500	150.0	Lower-middle income	-
	Nicaragua	20,918	177.6		-
	Haiti	7,523	302.0		-
	Anguilla	36	91.7	NI	-
	Guadeloupe	867	140.0		-
	Martinique	1,003	135.9		-
	Subtotal	322,919			
South America	Chile	57,048	91.3	High income	Suicide, alcohol and drug use, depression, psychosis, bipolar disorder, borderline, antisocial personality (5 articles)
	Guyana	2,156	151.0		-
	Uruguay	14,965	130.1		-
	Argentina	117,810	118.5	Upper-middle income	Alcohol and drug use (1 article)
	Brazil	839,672	173.9		Suicide, depression, drug use, stress, anxiety, psychosis, bipolar disorder (6 articles)
	Colombia	102,916	125.6		Suicide, depression, panic attack, PTSD, drug use, anxious (5 articles)
	Ecuador	33,106	119.5		Personality disorders, alcohol and drug use, depression, psychosis, anxiety (4 articles)
	Paraguay	17,712	171.6		-
	Peru	96,805	233.0		Depression (1 article)
	Suriname	1,000	75.2		-
	Bolivia	25,291	287.8	Lower-middle income	-
	French Guiana	1,022	165.9	NI	Antisocial personality, alcohol and drug use, anxiety, PTSD, depression, panic attack, dysthymia (2 articles)
	Venezuela	67,200	163.8		-
	Subtotal	1,376,703			

ADHD: attention deficit hyperactivity disorder; NI: no information; PTSD: post-traumatic stress disorder.

* Source: World Prison Brief ^85^;

** Source: World Prison Brief ^3^;

*** Source: Metreau et al. ^25^.

### Mental health disorders in the incarcerated population in the Americas

The reviewed articles used the concept of mental health disorders similar to the clinical definition, based on specific criteria established by diagnostic and statistical manuals of mental disorders. Mental disorders - which are characterized by significant changes in thought, mood, and behavior - can lead to distress or impairment in social, occupational, or other domains of life ^46^ ^,^ ^51^ ^,^ ^68^ ^,^ ^69^. Studies described the etiology of mental disorders as biopsychosocial, involving biological, psychological, and social factors, which helped to elucidate the impact of each country’s economic, cultural, and social conditions on the general population’s mental health ^43^, as well as the health impacts due to the circumstances of incarceration ^51^ ^,^ ^62^.

Overall, the selected studies reported a high prevalence of mental disorders among incarcerated populations compared to the general population. Similar themes were explored across the Americas (Box 1 details country-specific data), and a high rate of comorbid mental disorders was observed in the incarcerated population of Chile, notably among those with severe mental disorders, personality disorders, and psychoactive substance use ^50^. The study conducted in Grenada found a similar link between personality disorders and substance use, highlighting histrionic, antisocial, borderline, and aggressive-sadistic as the most prevalent disorders ^69^.

Another study conducted in Grenada revealed that over 60% of inmates experienced some type of mental illness, such as anxiety, depression, bipolar affective disorder, and schizophrenia ^49^. Depressive symptoms were prominently noted in studies of prison populations in Colombia, Chile, and Peru ^40^ ^,^ ^51^ ^,^ ^70^, with over half of the inmate populations surveyed showing signs of depression. Additionally, PTSD have a high prevalence in incarcerated populations ^43^. Only one study, conducted in Canada, explored cognitive deficits and found this diagnosis in a quarter of the incarcerated population studied ^47^.

Significant gender differences were observed regarding prevalence and manifestation of mental health disorders. Substance use, personality disorders, and attention deficit hyperactivity disorder (ADHD) were more common among men than women ^36^ ^,^ ^50^ ^,^ ^70^. One study, however, found similar rates of substance use between male and female inmates ^29^. A study conducted in Chile reported that the risk of suicide reached nearly one-third among incarcerated men, with higher prevalence than in the female population ^70^. Also, there is the triad of disorders in Chile: severe mental disorder, personality disorder, and substance use disorder were more common among young men with less education and were associated with previous incarceration and histories of psychiatric hospitalizations ^50^.

Research on the incarcerated population in Brazil has shown that women experience higher rates of mental illness compared to men. Anxiety disorders, affective disorders, and severe mental illnesses are more prevalent among women, which are nearly double than those found in men. Additionally, black and Indigenous women are identified as being more vulnerable to mental health issues ^30^ ^,^ ^71^. A study in Canada found that the most common disorders among convicted women included PTSD, major depression, antisocial personality disorder, substance use, and anxiety disorders ^30^. Among these, depression and anxiety were highlighted as predictors for suicide risk ^53^, particularly higher at the beginning of incarceration ^57^.

Studies from Ecuador and Brazil align with the aforementioned findings on the comparison between women and male inmates ^46^ ^,^ ^48^. Women are also more vulnerable to mental health disorders than both the general population and incarcerated men, owing to higher lifetime exposure to violence and limited coping resources ^48^. Furthermore, self-harm behavior is more prevalent among women, often associated with factors such as low educational attainment and financial difficulties ^58^ ^,^ ^63^.

The primary risk factors impacting the mental health of incarcerated individuals are related to: (i) length of incarceration, with a noted and casual decrease in depressive symptoms in the first year (although levels were so high on admission that almost half still fulfilled criteria for major depression after one year) ^57^; (ii) weak family ties, which contribute to mental health challenges ^41^ ^,^ ^46^; (iii) low emotional support, which is correlated with higher prevalence of anxiety disorders ^59^; (iv) lack of work and educational opportunities ^64^; (v) separation from children ^64^; (vi) history of traumatic events ^64^; (vii) overcrowded prisons, inadequate cell conditions, poor nutrition, a sense of lost control over one’s life, and inadequate medical attention ^46^; and (viii) substance use within the prison environment ^41^.

Mental health disorder is also linked to physical health issues. For instance, the prevalence of alcohol abuse disorders among incarcerated individuals with HIV in Argentina is five times higher than in the general population, which severely hinders the consistent treatment of HIV ^35^. Additionally, one-third of deaths in the Canadian prison system were associated with mental health issues ^31^.

### Mental disorders, violent behavior and criminal recidivism

Studies that associate the prevalence of mental disorders with criminal recidivism have been conducted in the United States (n = 1), Canada (n = 3), French Guiana (n = 1), and Ecuador (n = 1). Substance use has been linked to higher recidivism rates ^28^ ^,^ ^43^. Antisocial personality disorder was associated with the recurrence of violent offenses ^43^. Inmates with more than one mental disorder also showed higher recidivism rates ^68^. Additionally, evidence suggests inmates with high levels of ADHD symptoms return to prison shortly after release, indicating that ADHD may be a risk factor for recidivism ^36^.

Traumatic events in early life, such as emotional and sexual abuse, were common among individuals who were homeless, committed crimes, and were incarcerated. These experiences can lead to further exposure to violence, substance use, mental disorders, and homelessness, leaving individuals more vulnerable to incarceration and greater mental health challenges ^32^.

An investigation in Ecuador compared the profiles of recidivist and non-recidivist offenders, finding that recidivists showed traits of antisocial, borderline, and aggressive-sadistic personality, high levels of alcohol and other drugs dependence, increased aggression, juvenile delinquency, more disciplinary records in prison, and psychological problems prior to incarceration. According to this study, the types of crimes most associated with recidivism included property crimes, drug trafficking offenses, and, to a lesser extent, violent crimes ^61^.

While an publication from the United States ^28^ associated the study on mental disorders and criminal recidivism with a focus on effective prison practices aimed at reducing recidivism costs, the publications from Canada ^32^ ^,^ ^36^ ^,^ ^68^ and Ecuador ^61^ associated mental health care for the incarcerated population with rehabilitation, based on the legal provisions of their respective countries. The study on recidivism in Ecuador contributed to assessing the effectiveness of the penitentiary system and offering rehabilitation interventions for those whose mental disorders contribute to recidivism, aiming at reducing the prison population. This is aligned with the country’s current Federal Constitution, Criminal Code, and National Plan for Good Living (2013-2017 ^72^/2017-2021 ^73^), which establish that the State’s response to violence integrates police, justice, and social rehabilitation, rather than relying on the traditional reactive police response.

The restorative approach integrated in Canada’s penal execution system ^67^ seems to be strongly associated with a focus on the rehabilitation of violent offenders with mental disorders. Identifying the mental health needs of the prison population enables the proposal of appropriate interventions for this group ^36^, which assists in their social reintegration and reduces the incarceration of individuals whose violent behavior requires health and social assistance interventions ^32^ ^,^ ^74^ rather than punitive responses. Additionally, proposing more attentive mental health care for the incarcerated population can contribute both to improving their quality of life and to reducing the risk of criminal recidivism ^68^.

The economic factors of Ecuador (classified as an upper-middle-income country) and Canada (a high-income country) do not seem to be determinants in explaining the focus on social rehabilitation for incarcerated individuals. Instead, the cultural factors in both countries support a less punitive approach in State responses to violence.

A publication regarding French Guiana acknowledges that, although it is a French territory, it has an economic profile closer to low- and middle-income countries ^43^, where the prevalence of psychosis and depression is higher, along with poorer investment in mental health care for the prison population ^43^. In this publication, the authors recognize that penitentiaries and mental health services have different objectives, but they should not be antagonistic; because providing mental health care to the prison population can benefit both the affected individuals and public safety ^43^.

Regarding incarceration impact on the mental health of imprisoned individuals, it has been observed that incarceration exacerbates mental illness. Moreover, those with mental disorders are more susceptible to physical violence within prisons, which means that incarcerated individuals with mental disorders face a higher risk of victimization compared to the general population ^49^. A study in Mexico ^38^ concluded that overcrowded prisons were associated with higher rates of substance use. Individuals with a history of incarceration showed higher rates of PTSD compared to those who had not been incarcerated. Continuous exposure to trauma was identified as a common experience among formerly incarcerated individuals. Receiving visits and maintaining strong family ties were associated with lower levels of depression. Furthermore, working while serving a sentence was found to be protective against substance use ^38^.

### Mortality in the population deprived of liberty

One study from the United States ^56^ found that increases in county jail incarceration rates were associated with higher mortality rates for various causes of death. Specifically, a one per 1,000 increase in the incarceration rate was linked to significant increases in mortality from infectious diseases (6.5%), chronic lower respiratory diseases (4.9%), substance use (2.6%), and suicide (2.5%) among individuals younger than 75 years within one year of the increase.

Studies about mortality in the population deprived of liberty have been conducted in the United States (n = 1), Brazil (n = 1), French Guiana (n = 1), Colombia (n = 2) and Chile (n = 2). Suicide was the most frequent cause of death in the articles, and South America led the publications on suicide in prison on the continent ^27^ ^,^ ^34^ ^,^ ^37^ ^,^ ^44^ ^,^ ^53^ ^,^ ^55^, with emphasis on upper-middle income countries.

The risk of suicide was reported to be highest within the first few weeks of incarceration, which suggests that the adaptation may be a critical factor ^27^ ^,^ ^34^. The conditions of incarceration and the psychological state of inmates were identified as important factors influencing suicidal behavior. The lack of psychological support during incarceration limits the ability of the inmates to manage their emotional and mental health issues, making it a risk factor for suicide ^27^.

A study conducted among prisoners in French Guiana ^53^ identified a low suicide risk compared to France, which was associated with various factors, including a high proportion of foreign prisoners (due to international drug trafficking), who may lack strong community ties and potentially face less social stigma associated with incarceration. Another study in Colombia highlighted sociodemographic factors linked to increased suicidal ideation in prison: young individuals (aged 19 to 29), single, low educational attainment, lack of prison labor activities, from low socioeconomic backgrounds, and with a history of intrafamilial violence ^44^.

Mortality rates among individuals who are incarcerated or have been released from prison are higher compared to those without a history of incarceration and are related to violence, suicide, and infectious diseases. In a Brazilian study, while men are more likely to die from homicide, incarcerated women are more vulnerable to deaths from suicide and infectious diseases ^55^. On the other hand, a study from Colombia showed that women have higher rates of attempted suicide, but men are more successful with suicide ^34^. The risk of violent death and suicide was the highest immediately after the release from prison ^55^. The article suggests that mortality rates may be underestimated due to underreporting of deaths in custody and incomplete data regarding incarceration status at the time of death ^55^.

Motherhood is a protective factor against the risk of suicide in women deprived of liberty in Chile, which was associated with a sense of responsibility for their children. According to the researchers, in Latin American cultures, family relationships, especially between parents and children, are stronger than in Western cultures. Thus, the separation of children via incarceration can be perceived as very painful, which triggers mental suffering, but also protective against the risk of suicide, due to the moral responsibility towards one’s own children ^37^.

### Stigma as a barrier to accessing mental health services

Although the articles analyzed did not specifically aim to study stigma, its implications can be inferred from the conclusions and discussions surrounding mental health issues in prisons. Publications from upper-middle income and high-income countries addresses the topic similarly, however, studies from Latin America were more frequent in pointing out stigma as a barrier to access mental health services in prisons.

Incarcerated individuals suffering from mental illness may face dual stigma, with two primary social markers overlapping and further marginalizing this population: crime and mental illness ^33^ ^,^ ^42^ ^,^ ^61^. Additionally, intersecting factors of race/ethnicity, gender identity, and sexual orientation among prisoners with mental disorders can intensify the experience of stigma. For instance, incarcerated women with mental health issues may experience greater stigmatization due to societal expectations of women in a patriarchal context, especially regarding motherhood. This greater stigmatization can hamper their pursuit of necessary treatments ^30^ ^,^ ^33^ ^,^ ^56^ ^,^ ^64^.

The fear of being perceived as weak when discussing mental health disorders with fellow inmates or prison staff also increases avoidance of treatment, worsening individuals’ mental health ^38^ ^,^ ^41^ ^,^ ^50^ ^,^ ^58^.

Stigma is also noted as a factor that negatively affects the self-reporting of mental health assessments by prisoners, as they may not recognize their mental health condition or may fear repercussions if they disclose symptoms, such as transfer to a psychiatric facility ^29^ ^,^ ^51^. Such factors impact the knowledge about the mental health realities of the incarcerated population, which is essential for grounding and planning effective mental health intervention policies.

The stigma associated with mental disorders within the prison context impacts the quality of healthcare services provided, as being deprived of liberty limits the capacity of the individuals to act and seek help on their own. Access to care often depends on intermediaries, such as staff who may lack proper training to understand the complexities involved in mental health conditions ^58^, which can contribute to increased mortality among incarcerated individuals ^31^.

The negative perception of society of the incarcerated population shapes rehabilitation and treatment programs and may influence public policy. This perspective may lead to a lack of political support and underfunding for mental health services in prisons, creating barriers to social reintegration efforts ^45^. Increasing mental health awareness and education for both inmates and prison staff can help reduce stigma, expanding access to necessary treatments by mitigating fears of judgment and further stigmatization ^31^ ^,^ ^32^ ^,^ ^38^ ^,^ ^41^ ^,^ ^42^ ^,^ ^50^ ^,^ ^58^ ^,^ ^71^.

Finally, the development of policies for mental health and substance use treatment for incarcerated individuals should incorporate strategies to address and lessen the negative impact of the stigma associated with seeking treatment, thus enhancing the overall effectiveness of mental health interventions in prisons ^33^ ^,^ ^37^ ^,^ ^38^.

## Discussion

This study aimed to conduct a scoping review of scientific literature on the presence of mental disorders in the prison population in American countries, based on a global mental health perspective. South American countries were the most investigated, followed by North American countries. Only two of the 42 studies selected were conducted in Central America. The limited literature on the mental health of the prison population in Central America contrasts with the data on incarceration in the region: Central America has the highest incarceration rate globally ^1^ and the largest gap in mental health treatments ^16^. The punitive context surrounding prisons can overshadow the extent of mental health care demanded by prisoners, who have a higher prevalence of mental disorders than the general population ^7^ ^,^ ^8^ ^,^ ^10^ ^,^ ^75^.

Mental disorders investigated in prisoners in the Americas were as follows: use of alcohol and drugs, PTSD, depression, anxiety, panic attack, dysthymia, ADHD, psychosis, personality disorder, cognitive deficit, bipolar disorder, self-injury, and suicide. Cognitive deficit was investigated only in a study from Canada, a high-income country and ranks at the 4th position in the world for education system ^76^. There is no information about cognitive deficits in prisoners from other countries in the American continent, especially Latin America and the Caribbean that have poor levels of overall education ^77^.

Studies have identified different prevalence rates of mental disorders among men and women, and they show that women have higher rates of mental illness. Risk factors for mental illness in incarcerated individuals involve the length of time people are deprived of liberty, in which the beginning of serving a sentence is a period of greater illness and risk of suicide ^27^ ^,^ ^34^ ^,^ ^57^, the weakening of family ties ^41^ ^,^ ^46^, lack of work and study opportunities ^64^, separation of children ^64^, history of experiencing traumatic events ^64^, overcrowded prisons ^46^, and substance use within prison ^41^. The conditions of incarceration and the psychological state of inmates were important factors influencing suicidal behavior ^27^.

Mental health disorders were also related to criminal recidivism ^28^ ^,^ ^36^ ^,^ ^43^ ^,^ ^68^, and approaches focused on rehabilitation and mental health care have been more effective in social reintegration and reducing recidivism than traditional punitive models ^32^ ^,^ ^36^ ^,^ ^61^ ^,^ ^68^. While there are significant treatment gaps for the incarcerated population in need of mental health care ^14^ ^,^ ^15^, there are also a gap of different treatments between Americas: the gap in Latin America is larger than Northern America ^16^. Countries in the Global South often face significant economic challenges, including high levels of poverty, unemployment, and inequality, which can contribute to mental health disorders, making it essential to consider the broader context when addressing mental health care ^78^.

Notably, differences in the mental health of incarcerated people vary across countries with different income levels due to factors such as health infrastructure, resource availability, and social determinants. We highlight the following aspects: (i) access to mental health services which, although insufficient, are more common in high-income countries; (ii) the prevalence of mental disorders is another aspect, showing higher rates of untreated mental disorders due to inadequate services and the compounded effects of inequality, poverty, and violence in middle/low-income countries; (iii) the quality of care offered also differs, in which wealthier countries tend to offer a higher quality of mental health care, with access to medications and therapies; (iv) stigma and social factors are found in all countries, but are more severe in low- and middle-income countries and can further discourage prisoners from seeking care, and cultural factors often exacerbate these issues; (v) the impact of overcrowding, which is greater in countries with less wealth ^79^ ^,^ ^80^.

Distinguishing between the Global North and the Global South when discussing global mental health involves the understanding of how socioeconomic, cultural, and historical conditions affect access to and approach to mental health treatment in such distinct contexts. The WHO and the Movement for Global Mental Health (MGMH) significantly shape mental health policies by providing frameworks, advocating for global priorities, promoting standardized practices, and influencing funding decisions. However, there is a growing recognition of the need to critically assess such influences to ensure that mental health policies are culturally relevant and effective in addressing the unique challenges faced by communities in low- and middle-income countries ^81^ ^,^ ^82^ ^,^ ^83^. Frameworks and policies promoted by the WHO and MGMH may not always align with local needs and realities, and there is a call for a more nuanced approach that incorporates local knowledge and practices ^81^. Addressing the structural disparities and cultural and historical contexts that distinguish the experiences of the Global North and the Global South is important for a full understanding of global health, encouraging a decolonial approach that prioritizes respect and integration of local needs ^82^.
